# Disease-associated alleles in genome-wide association studies are enriched for derived low frequency alleles relative to HapMap and neutral expectations

**DOI:** 10.1186/1755-8794-3-57

**Published:** 2010-12-10

**Authors:** Joseph Lachance

**Affiliations:** 1 Graduate Program in Genetics, Department of Ecology and Evolution, Stony Brook University, Stony Brook, NY 11794-5222; 2 Department of Genetics, 430 Clinical Research Building, 415 Curie Blvd., University of Pennsylvania, Philadelphia, PA 19104-6145

## Abstract

**Background:**

Genome-wide association studies give insight into the genetic basis of common diseases. An open question is whether the allele frequency distributions and ancestral vs. derived states of disease-associated alleles differ from the rest of the genome. Characteristics of disease-associated alleles can be used to increase the yield of future studies.

**Methods:**

The set of all common disease-associated alleles found in genome-wide association studies prior to January 2010 was analyzed and compared with HapMap and theoretical null expectations. In addition, allele frequency distributions of different disease classes were assessed. Ages of HapMap and disease-associated alleles were also estimated.

**Results:**

The allele frequency distribution of HapMap alleles was qualitatively similar to neutral expectations. However, disease-associated alleles were more likely to be low frequency derived alleles relative to null expectations. 43.7% of disease-associated alleles were ancestral alleles. The mean frequency of disease-associated alleles was less than randomly chosen CEU HapMap alleles (0.394 vs. 0.610, after accounting for probability of detection). Similar patterns were observed for the subset of disease-associated alleles that have been verified in multiple studies. SNPs implicated in genome-wide association studies were enriched for young SNPs compared to randomly selected HapMap loci. Odds ratios of disease-associated alleles tended to be less than 1.5 and varied by frequency, confirming previous studies.

**Conclusions:**

Alleles associated with genetic disease differ from randomly selected HapMap alleles and neutral expectations. The evolutionary history of alleles (frequency and ancestral vs. derived state) influences whether they are implicated in genome-wide assocation studies.

## Background

The onset of affordable high-throughput genotyping technology has enabled association studies to be conducted on a genome-wide scale, and multiple successes have occurred using this approach [1,2]. Notable examples include genes associated with LDL cholesterol levels [3], colorectal cancer [4], and type 1 diabetes [5]. Despite these successes, genome-wide association studies (GWAS) have been unable to account for the majority of heritable variation for most diseases [6,7]. One reason for the mixed success of GWAS is that the efficacy of such studies depends upon the underlying genetic architecture of traits [8-10]. Also relevant is the accuracy of the common disease-common variant hypothesis. Under this formulation, complex diseases are caused by high frequency alleles. By contrast, the genetic heterogeneity (rare allele-major effect) hypothesis proposes that distinct low-frequency alleles are responsible for the same trait in different individuals. Regardless of the relative validity of each of these hypotheses [11,12], there are now enough genome-wide association studies to be able to say something about the frequencies and ancestral or derived state of disease-associated alleles relative to HapMap and neutral expectations. A previous study gave an initial glimpse into the nature of disease-associated alleles, finding a median allele frequency of 0.40 [13]. However, less than 50 replicated SNPs were analyzed in the review article by Iles. Multiple studies have found that the allele frequencies of disease-associated SNPs do not significantly differ across populations compared to random SNPs [14,15]. Additionally, alleles associated with genetic disease are underrepresented in intergenic regions and overrepresented in nonsynonymous sites and 5 kb promoter regions [16]. An open question is whether the allele frequency distribution and ancestral vs. derived states of disease-associated alleles differ from the rest of the genome. What types of disease-associated alleles have been found in GWAS to date?

Alleles can be classified by frequency, ancestral vs. derived state, and relative disease risk. Disease-associated marker alleles need not be causal; they may be linked to alleles that are actually responsible for increased risk. Importantly, the alleles characterized in this paper are marker alleles. Because statistical power is maximized at intermediate allele frequencies [17], alleles detected in a GWAS are unlikely to be rare. Alleles can also be classified as ancestral or derived, where ancestral alleles are shared with closely related species and derived alleles are not [18]. There is currently a lack of published studies indicating whether disease-associated alleles are enriched for ancestral or derived states. Finally, alleles can be classified by their relative risk (measured as an odds ratio). In many GWAS, disease-associated alleles increase risk by only a modest amount, i.e. odds ratios are less than 1.5 [1]. However, it is unknown whether these odds ratios vary by ancestral vs. derived state. In addition, the first study to detect a particular association can overestimate the odds ratio, a phenomenon dubbed the "winner's curse" [19].

The aim of this study was to determine the characteristics of disease-associated alleles and compare this data to null expectations from HapMap data and the neutral theory. Allele frequency distributions and ancestral vs. derived states were obtained for every disease-associated allele found in genome-wide association studies prior to January 2010. The hypothesis that disease-associated alleles do not differ from the rest of the genome [20] was also tested.

## Methods

### Null expectations: HapMap data

Alleles from the HapMap dataset were obtained to serve as a baseline of genomic diversity. These alleles were subsequently weighted by the probability of detection in a GWAS. The set of all HapMap SNPs from data release #27 were downloaded via the HapMart tool [21]. This build included merged data from phases II and III of the International HapMap Project [22]. A Perl script was then used to randomly select 1000 unique SNPs from this file. The positions of HapMap SNPs were tested to ensure that linkage disequilibrium was minimal (distances between SNPs were at least 200 kb). Because allele frequencies can vary from population to population, and the majority of GWAS use European and European-American cases and controls, CEU allele frequencies were used in this study. This allowed HapMap dataset to act as a control for demographic processes. While disease prevalence and allele frequencies vary among populations [23], population-level differences in allele frequencies are similar for disease-associated SNPs and random genomic SNPs [14]. An additional consideration is that SNP discovery protocols may bias the HapMap dataset towards high frequency alleles [24,25]. For each SNP, alleles were chosen at random after weighting by allele frequency. Thus, a SNP with allele frequencies of 0.80 and 0.20 would have an 80% chance of yielding the major allele. Because high frequency alleles are more likely to be ancestral [26], randomly chosen alleles from the HapMap dataset are more likely to be ancestral than derived.

Outgroups (such as chimpanzees) enable SNPs to be polarized and ancestral states to be inferred. Ancestral alleles were determined via BLAST searches of disease-associated SNP regions against the chimpanzee genome. In addition, the single nucleotide polymorphism database, dbSNP, contains information on the ancestral state of SNPs at http://www.ncbi.nlm.nih.gov/projects/SNP[27]. Ancestral alleles in dbSNP were inferred via parsimony [28]. SNPs were only selected if ancestral allele states could be inferred. HapMap alleles were then binned by allele frequency and ancestral vs. derived state. Unlike disease-associated alleles, randomly selected HapMap alleles are not weighted by their probability of detection. Because of this, comparisons between the allele frequency distribution of disease-associated alleles and null expectations incorporated the probability that a particular allele is detectable in a GWAS. Phase three of the HapMap project used the Illumina Human1 M and Affymetrix SNP 6.0 platforms (the same platforms used in many GWAS). This indicates that the HapMap dataset served as an appropriate control for GWAS data.

### Null expectations: neutral theory

Population genetic theory was used to test whether disease-associated SNPs differ from neutral expectations. These expectations were subsequently weighted by the probability of detection in a GWAS. An infinite sites model was used to obtain the theoretical allele frequency distribution of neutral loci. Under this model, novel mutations occur at different nucleotide sites [29]. Marker loci were assumed to be diallelic and lack recurring mutations. In constant sized populations the probability of observing a derived neutral allele at a particular frequency is inversely proportional to allele frequency [30]. The parameter *C *in the equations below is a normalizing constant, and "unweighted" refers to the fact that these probability density functions do not incorporate the probability of detection in a GWAS. *x *is the frequency of the disease-associated allele at a marker locus.

(1) $$P(x\text{=}X\text{, unweighted}|\text{derived})=\frac{C}{x}$$

(2) $$P(x\text{=}X\text{, unweighted}|\text{ancestral})=\frac{C}{1-x}$$

**Figure 1:**
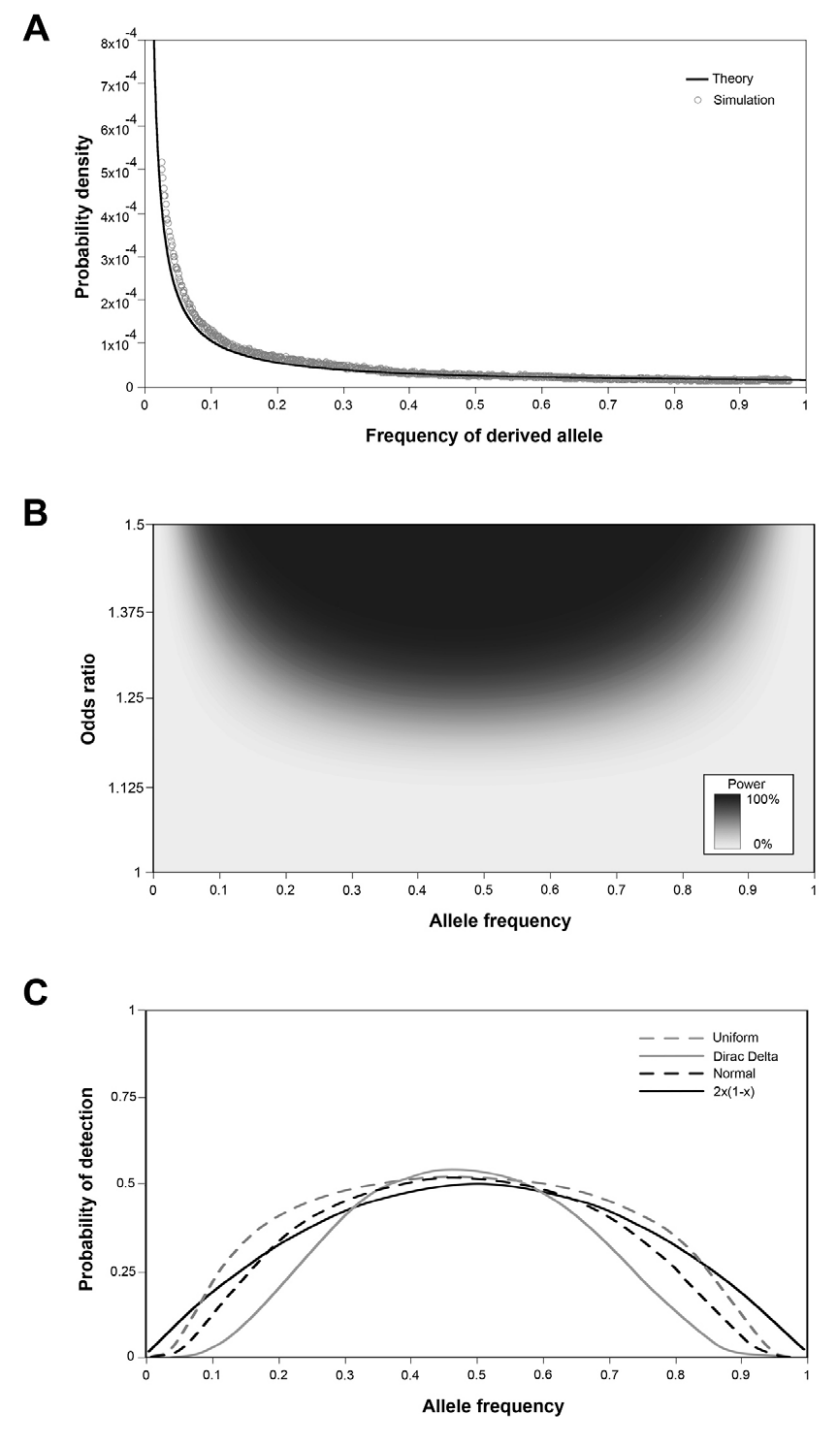
**Neutral expectations, statistical power, and probability of detection**. A) Unweighted probability density of derived allele frequencies under the neutral theory. Calculations use Equation 1 and are represented by a black line. MATLAB simulation data is represented by grey circles. B) Statistical power of GWAS plotted as a function of odds ratio and allele frequency. Darker shading indicates greater statistical power. Parameter values: 2500 cases and controls, a *p*-value of 5 × 10^-8^, complete linkage, multiplicative dominance. C) Probability of detecting a disease-associated allele. Three distributions of odds ratios are considered: uniform (evenly distributed odds ratios between 1.0 and 1.5, dashed grey line), Dirac delta (every allele has an odds ratio of 1.25, solid grey line), and normal (mean 1.25, standard deviation = 0.1, dashed black line). Equation 6 is plotted as a solid black line.

The probability density of Equation 1 goes to infinity as allele frequencies go to zero. Because of this, polymorphisms were only considered if the minor allele frequency was above some arbitrary threshhold frequency, *d*. Allele frequencies were allowed to range from *d *to 1-*d*, with the parameter *d *arbitrarily set equal to 0.025. MATLAB [31] simulations verified the accuracy of Equation 1 (see Figure 1A). In these simulations, alleles were binomially sampled each generation and frequencies of derived alleles were recorded. Upon fixation or loss, a single derived allele was allowed to enter the population. Simulations were run for 10^7 ^time steps with a population size of 10^4 ^individuals.

The probability that an allele is ancestral is equal to its probability of fixation [32,33]. For neutral loci, the probability that a randomly chosen allele is ancestral is simply its allele frequency [26].

(3) P(ancestral|x=X)=x

The right-hand sides of Equations 2 and 3 were multiplied and integrated from *d *to 1-*d*. This expression was then normalized by dividing by the total probability density, giving the overall probability that a neutral allele is ancestral (unweighted by the chance of detection in a GWAS).

(4) $$P\text{(ancestral, unweighted)}=\frac{\int_{d}^{1-d}\frac{x}{\text{1-x}}\;dx}{\int_{d}^{1-d}\frac{1}{\text{1-x}}\;dx}$$

After integration and extensive algebra:

(5) $$P\text{(ancestral, unweighted)}=1+\frac{2d-1}{\ln(1-d)-\ln(d)}$$

### Probability of detection (statistical power calculations)

Because statistical power varies by allele frequency [17], computer simulations were used to calculate the probability of detecting an association between disease and a marker allele at a particular frequency. Statistical power calculations were obtained via the Windows program QUANTO [34,35]. This program numerically calculates power for a variety of experimental designs, but it does not explicitly take into account ages of SNPs and ancestral vs. derived states. Statistical power is a function of linkage disequilibrium between causal and marker alleles (see Appendix). The following parameter values were used: 2500 cases and controls, a *p*-value of 5 × 10^-8^, complete linkage, multiplicative dominance. Statistical power was calculated for odds ratios ranging from 1 to 2 (at increments of 0.01) and allele frequencies ranging from 0 to 1 (at increments of 0.01). As indicated by Figure 1B, alleles with low odds ratios can only be detected at intermediate allele frequencies.

Although the true distribution of odds ratios for the set of all disease-associated alleles is unknown, most disease-associated alleles increase relative risk by small amount (i.e. odds ratios are less than 1.5) [1]. Three different distributions of odds ratios were considered: uniform (evenly distributed odds ratios between 1.0 and 1.5), Dirac delta (every allele has an odds ratio of 1.25), and normal (mean 1.25, standard deviation = 0.1). Plots of statistical power vs. allele frequency were similar for each of these distributions (Figure 1C). A simple expression yields a reasonable estimate of statistical power given the parameter values listed above. For mathematical simplicity, subsequent calculations assume that the probability of detecting a disease-associated allele is:

(6) P(detection|x=X, parameter values listed above)≈2x(1−x)

It is coincidental that the expression in Equation 6 also gives the expected heterozygosity. Different sample sizes and/or odds ratios would yield different expressions for the probability of detection.

### Null expectations: weighted frequency distributions

HapMap and neutral expectations were weighted by the probability of detection to enable fair comparisons with disease-associated alleles. Probability densities of each allele frequency bin were multiplied by the expression in Equation 6 and normalized. This resulted in elevated probabilities of observing intermediate frequency alleles. For neutral expectations.

(7) P(x=X, weighted|derived)=2(1−x)

(8) P(x=X, weighted|ancestral)=2x

The right-hand sides of Equations 3 and 8 were multiplied and integrated from *d *to 1-*d*. This expression was then normalized by dividing by the total probability density, giving the overall probability that a neutral allele is ancestral (weighted by the chance of detection in a GWAS).

(9) $$P\text{(ancestral, weighted)}=\frac{\int_{d}^{1-d}{\text{2x}}^{\text{2}}\;dx}{\int_{d}^{1-d}\text{2x }dx}$$

After integration and extensive algebra:

(10) $$P\text{(ancestral, weighted)}=\frac{2}{3}(d^{2}-d+1)$$

### Empirical data: disease-associated alleles

The set of all disease-associated alleles found prior to January 1, 2010 was obtained to investigate whether these alleles differed from the rest of the genome. An excellent database of GWAS and disease-associated SNPs exists online at http://www.genome.gov/gwastudies and it was used in this study [16,36]. This Catalog of Published Genome-Wide Association Studies includes every disease-associated SNP to date. Criteria for inclusion in this database included *p*-values < 10^-5 ^and a minimum of 100,000 SNPs tested in the initial stage of a study [16]. The set of all genome-wide association studies prior to January 1, 2010 spans 486 papers and a total of 2186 disease-associated SNPs. Some of these SNPs were present in multiple studies, and in many cases the disease-associated allele was not listed in the database. Allele frequencies in control populations were obtained from NHGRI's Catalog of Published Genome-Wide Association Studies. When allele frequency data were absent from this database, CEU HapMap frequencies were used. If a particular SNP was associated with diseases in multiple studies, mean allele frequencies were calculated. The ancestral vs. derived state of each allele was determined via dbSNP. When ancestral allele state could not be inferred, SNPs were omitted from the dataset. After taking into account ancestral vs. derived states of alleles, a total of 1143 disease-associated SNPs remained. Of these SNPs, 530 had odds ratio data.

For comparisons with null expectations, disease-associated SNPs were binned into 10% allele frequency intervals (see Figure 3). Differences between the allele-frequency distributions of disease-associated alleles and null expectations were assessed via χ^2 ^goodness-of-fit tests. Relative magnitudes of disease-associated and control allele frequencies were compared via Mann-Whitney U tests. Proportions of ancestral alleles were compared via binomial tests, with a null hypothesis that equal proportions of disease-associated and control alleles were ancestral. In addition, 95% confidence intervals of mean allele frequency were found by sampling with replacement. This bootstrap analysis was performed in MATLAB (100000 replicates) [31]. It is possible that alleles found in multiple studies have different characteristics than alleles found in a single study. Because of this, the mean frequency and evolutionary history of replicated alleles (alleles implicated in multiple studies) were compared with the overall patterns of disease-associated alleles. A total of 142 replicated alleles had frequency and evolutionary history data.

Alleles implicated in GWAS were also sorted into seven different phenotypic classes (cancer, cardiovascular, metabolism, miscellaneous disease, morphological, and neurological). Some alleles were associated with multiple phenotypic classes. The morphological class included alleles that were not technically associated with any disease. Instead, they were associated with traits such as height and hair color. 92 of 1143 GWAS alleles were implicated in studies of non-European populations, and the remaining 1051 alleles were re-analyzed to determine whether this had any effect. Because of the small number of alleles implicated in studies of non-European populations, additional analysis was not conducted on these 92 GWAS alleles. See Additional file 1 for a list of SNPs, allele frequencies, ancestral vs. derived states, odds ratios, and phenotypic class.

To test whether the properties of disease-associated alleles changed over time, disease-associated were binned into six-month intervals by date of first publication. Mean frequency, probability ancestral, and odds ratio data were calculated for each time interval. To determine whether genotyping platforms biased the properties of alleles, data from Affymetrix, Illumina, and Perlegen arrays were compared. Many studies used multiple genotyping platforms, and characteristics of disease-associated alleles in these studies were also analyzed. The number of genotyped SNPs passing quality control varied in each study. To assess whether this had any effect, disease-associated alleles were binned into three intervals corresponding to the number of genotyped SNPs ( < 500,000, between 500,000 and 1,000,000, and >1,000,000). These numbers include imputed SNPs.

### Estimated ages of SNPs

Ages of SNPs were calculated to determine if detectable associations occur more often in young or old SNPs. Estimates of SNP ages were calculated from the allele frequency distributions of HapMap and GWAS alleles. Under the neutral theory [37], the expected age of a SNP (τ) is

(11) $$E(\tau )=\frac{-2x}{1-x}\ln(x)$$

*x *in Equation 11 refers to the frequency of the derived allele and time is measured in units of 2N_e _generations (where N_e _is the effective population size). SNPs with low frequency derived alleles tend to be younger SNPs. However, variance in τ tends to be quite large and allele frequencies only give a rough estimate of the SNP age. Because of this, the cumulative probability density function [38,39] was used.

(12) $$P\text{(}\tau \leq \text{t)}≅{\left(1-x\right)}^{-1+n/(1+nt/2)}$$

**Figure 2:**
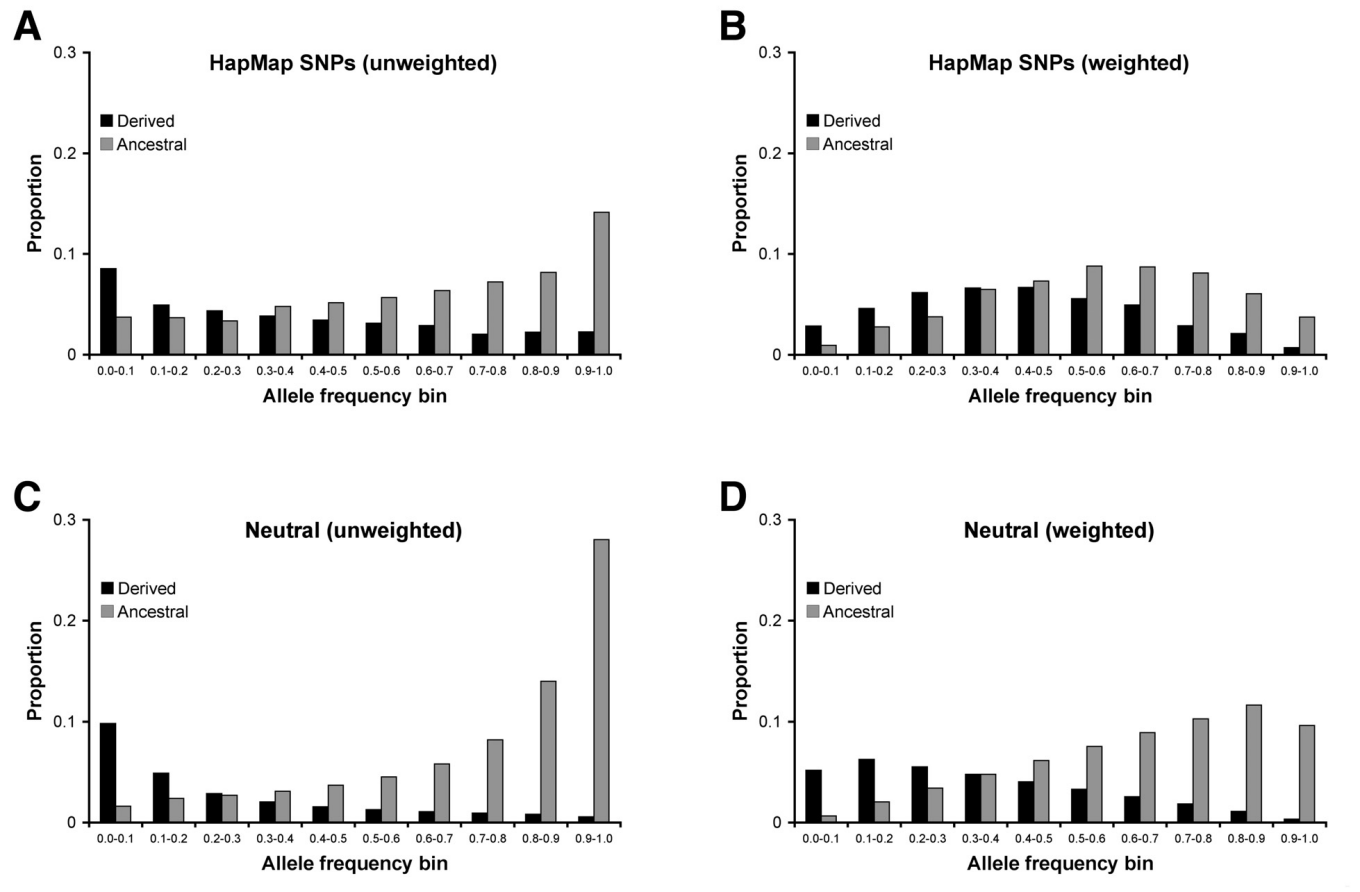
**Allele frequency distributions for null expectations**. Allele frequencies are binned into 0.10 intervals. Derived probabilities are labelled in black and ancestral probabilities are labelled in grey. Neutral expectations use a polymorphism threshold (*d*) of 0.025. A) Allele frequency distributions for HapMap alleles prior to detection (n = 1000). B) Allele frequency distributions for HapMap alleles after weighting by the probability of detection (n = 1000). C) Theoretical allele frequency distributions for neutral alleles prior to detection. D) Theoretical allele frequency distributions for neutral alleles after weighting by the probability of detection.

**Figure 3:**
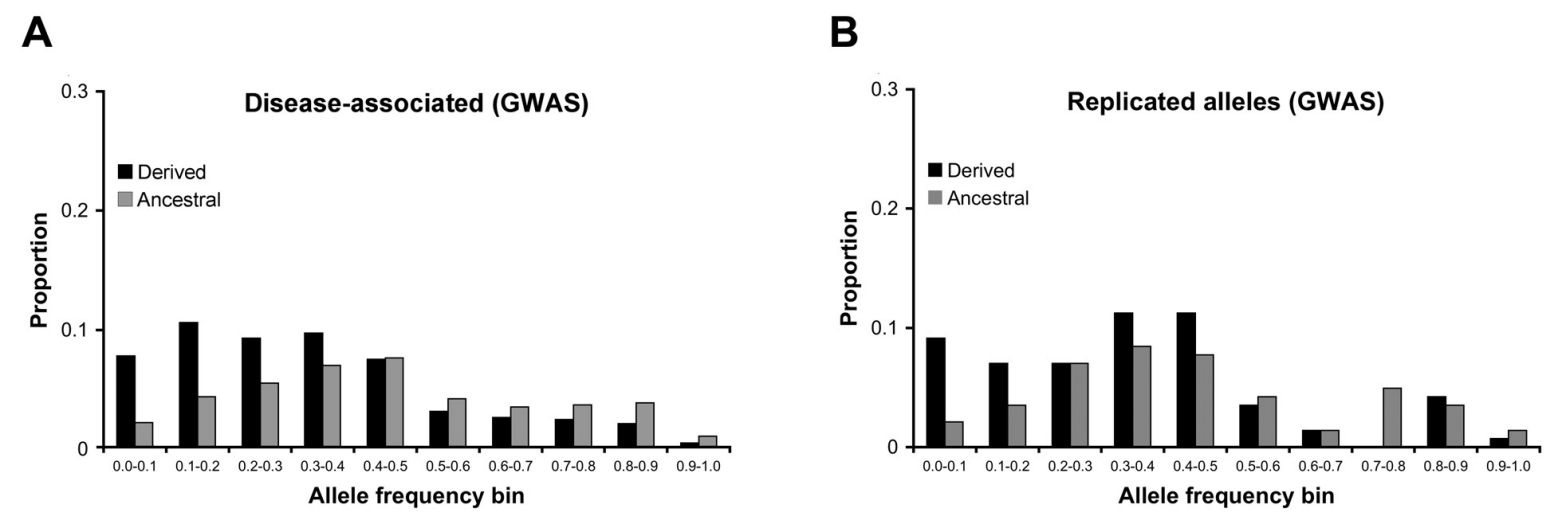
**Allele frequency distributions for GWAS data**. Allele frequencies are binned into 0.10 intervals. Derived probability densities are labelled in black and ancestral probability densities are labelled in grey. A) All disease-associated alleles (n = 1143). B) Disease-associated alleles that have been implicated in multiple studies (n = 142).

Sample size (*n*) in Equation 12 was arbitrarily set equal to 2500. The derivative of Equation 12 with respect to *t *was taken for a range of allele frequencies (0.05 to 0.95 at intervals of 0.10) and SNP ages (0 to 8 N_e _generations at intervals of 0.04 N_e _generations). Allele frequency distributions in Figures 2B and 3A were then used to generate the expected distributions of SNP ages for weighted HapMap loci and GWAS loci.

## Results

### Null expectations

HapMap alleles were qualitatively similar to neutral alleles. In both cases, randomly selected alleles were likely to be ancestral and high frequency (see Table 1). High frequency (0.80-1.00) ancestral alleles and low frequency (0.00-0.10) derived alleles were the most common types of alleles for neutral and HapMap datasets (Figure 2). Unweighted proportions of ancestral alleles were comparable to estimates from whole-genome data (0.707 for a French individual) [40]. Weighting by the probability of detection in a GWAS increased the probability of observing intermediate frequency alleles and decreased the probability that an allele was ancestral.

**Table 1:** Disease-associated alleles vs. null expectations

	Mean frequency of a randomly chosen allele	Proportion ancestral
**Null expectations**		
HapMap data (n = 1000, unweighted)	0.721	0.623
HapMap data (n = 1000, weighted)	0.610	0.568
Theoretical (neutral, unweighted)	0.741	0.741
Theoretical (neutral, weighted)	0.650	0.650
**Disease-associated alleles**		
Cancer (n = 112)	0.362**	0.446*
Cardiovascular (n = 145)	0.364**	0.379**
Metabolism (n = 160)	0.365**	0.456*
Miscellaneous disease (n = 290)	0.413**	0.434**
Morphological (n = 276)	0.409**	0.467*
Neurological (n = 135)	0.429**	0.430*
Multiple phenotypic classes (n = 25)	0.312*	0.320*

**All GWAS alleles (n = 1143)**	0.394**	0.437**
**All replicated GWAS alleles (n = 142)**	0.396**	0.437**

Unweighted values do not incorporate the probability of detection, and weighted values incoporate the probability of detection in a GWAS. Neutral expectations use a polymorphism threshold (*d*) of 0.025 and Equations 5 and 10. Relative magnitudes of disease-associated and control allele frequencies were compared via Mann-Whitney U tests, and proportions of ancestral alleles were compared via binomial tests. * indicates significant differences from HapMap and neutral scenarios (*p*-value < 0.05), and ** indicates highly significant differences from HapMap and neutral scenarios (*p*-value < 0.0001). Totals for GWAS alleles are labeled in boldface type. Replicated alleles are those alleles that have been implicated in multiple studies.

Despite these similarities, there were important quantitative differences between HapMap alleles and neutral expectations. HapMap alleles were less likely to be ancestral than alleles under the neutral theory (0.623 vs. 0.741 for the unweighted scenario, and 0.568 vs. 0.650 for the weighted scenario). HapMap alleles were also more likely to be found at intermediate frequencies, and goodness-of-fit tests indicate that differences existed between the allele frequency distributions of HapMap and neutral alleles (*p*-value < 10^-10 ^for both the unweighted and weighted scenarios, χ^2 ^test with 19 d.f.). The relative lack of HapMap loci with a minor allele frequency < 0.1 may be due to ascertainment bias, as SNPs identified from a small panel of individuals are known to have an excess of intermediate frequency alleles [25].

Sensitivity analysis of the HapMap dataset suggests the absence of selection bias in the 1000 randomly selected SNPs. The mean frequency of minor alleles was 0.219 for the HapMap dataset used in this paper, compared to 0.214 and 0.199 for additional sets of 1000 SNPs from the HapMap (CEU) and Perlegen (EUR) databases, respectively.

### Disease-associated alleles

Empirical data from genome-wide association studies indicated that a majority of disease-associated alleles were derived. Out of 1143 unique SNPs, disease-associated alleles were ancestral in 499 cases and derived in 644 cases. As shown in Table 1 the proportion of ancestral alleles was less than HapMap and neutral theory expectations (*p*-value < 0.0001, binomial test for each comparison). Alleles shared with chimpanzees were less likely to be associated with genetic disease than alleles that are not shared with chimpanzees.

The majority of disease-associated alleles had frequencies below 0.50 (Figure 3). Goodness of fit tests indicated that the empirical allele frequency distribution differed from both the HapMap and neutral expectations (*p*-value < 0.0001 in both cases, χ^2 ^test with 19 d.f.). Allele frequency bins containing the highest proportion of disease-associated alleles were derived alleles with frequencies between 0.00 and 0.50 and ancestral alleles with frequencies between 0.30 and 0.50. The mean allele frequency of disease-associated alleles was 0.394, and comparisons disease-associated alleles had lower frequencies than HapMap and neutral expectations (*p*-value < 0.0001, Mann-Whitney U test). This is consistent with population genetics theory that predicts neutral variants linked to deleterious alleles should be found at lower frequencies [41]. 95% bootstrap confidence intervals of mean allele frequency ranged from 0.3802 to 0.4076. When data from studies of non-European populations were excluded, patterns were largely unchanged (mean frequency was 0.397 and the proportion of ancestral alleles was 0.428). Observed patterns were largely insensitive to *p*-value thresholds of GWAS: the correlation between allele frequency and negative log *p*-value was -0.0733, and disease-associated alleles found at *p*-values < 10^-8 ^had a mean frequency of 0.404. Mean allele frequencies for the complete set of GWAS alleles were 0.463 for ancestral and 0.340 for derived alleles, indicating that disease-associated alleles tended to be minor alleles. This is in contrast to null expectations, where ancestral alleles tended to be major alleles. The shape of the disease-associated allele frequency distribution did not resemble either null expectation, suggesting that additional factors were involved. Misidentification of ancestral states can result in an excess of high frequency alleles [42]. Because the dataset of disease-associated alleles had a deficiency of high-frequency alleles, this suggests that ancestral states were correctly inferred.

Alleles implicated in multiple studies showed similar patterns to the overall set of disease-associated alleles. Replicated alleles had a mean frequency of 0.396 and a 0.437 probability of being ancestral. The allele frequency distribution of replicated alleles also exhibited an excess of rare alleles relative to null expectations (Figure 3B).

**Figure 4:**
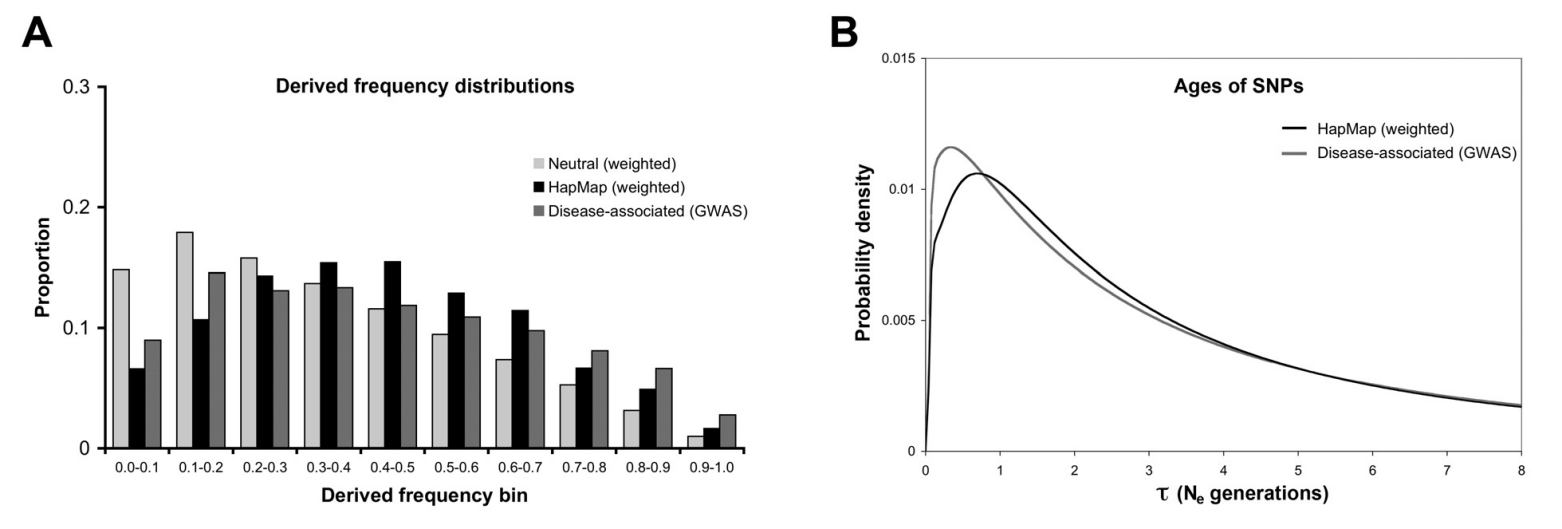
**Characteristics of different types of loci**. A) Derived frequency distributions. Derived allele frequencies are binned into 0.10 intervals and probability densities for different types of loci are indicated by shading (neutral expectations in light grey, HapMap SNPs in black, and disease-associated GWAS SNPs in dark grey). Similar numbers of loci were analyzed for each data type (1000 for neutral expectations and HapMap SNPs, and 1143 for GWAS SNPs). B) Estimated ages of SNPs. The probability density for HapMap SNPs (weighted by probability of detection) is labelled black and the probability density for disease-associated GWAS SNPs is labelled dark grey. Probability densities were obtained via Equation 12, and calculated at intervals of 0.04 N_e _generations.

Differences between disease-associated alleles and the rest of the genome can be due either to properties of loci or properties of alleles. Characteristics of loci were revealed in derived frequency distributions (Figure 4A). By contrast, the frequency distributions in (Figures 2 and 3) revealed characteristics of both alleles and loci. Goodness of fit tests indicated that derived frequency distributions differed for neutral expectations, HapMap SNPs, and disease-associated SNPs (*p*-value < 0.0001 for each pairwise comparison, χ^2 ^tests with 9 d.f.). However, derived frequency distributions were more similar than allele frequency distributions (compare Figures 2, 3, and 4). In addition, disease-associated SNPs and weighted HapMap SNPs had similar mean derived frequencies (0.426 vs. 0.432). This suggests that much of the difference between disease-associated alleles and the rest of the genome was due to properties of alleles rather than loci.

### Different phenotypic classes

Similar patterns were observed for each of the phenotypic classes (Table 1). In each case, disease-associated alleles had lower frequencies than HapMap and neutral expectations (*p*-value < 0.0001 for each comparison, Mann-Whitney U test). Regardless of phenotypic class, disease-associated alleles were more likely to be derived alleles than randomly selected HapMap alleles and neutral expectations (*p*-value < 0.05 for each comparison, binomial test). Alleles associated with cardiovascular disease were most likely to be low frequency derived alleles. Alleles associated with neurological disease had the highest mean allele frequency, and alleles associated with morphological traits were more likely to be ancestral. However, differences among phenotypic classes were smaller than the differences between each phenotypic class and null expectations (*p*-value < 0.05 for allele frequency and ancestral vs. derived data, One-way ANOVA).

### Additional controls

Characteristics of disease-associated alleles were independent of publication date and genotyping platform. Mean allele frequencies for each six-month interval were between 0.363 and 0.421. Similarly, the proportion of ancestral alleles ranged from 0.414 to 0.481. Median odds ratios for each six-month interval were between 1.24 to 1.365. Temporal trends were not observed for any of these characteristics. Allele frequencies of disease-associated alleles and proportion of ancestral alleles were similar for different genotyping platforms (Table 2). Median odds ratios were also similar for each manufacturer (1.28 for Affymetrix, 1.26 for Illumina, and 1.425 for Perlegen). Although the number of genotyped SNPs did not appear to affect mean allele frequency, the proportion ancestral alleles was slightly less for studies with >1,000,000 genotyped SNPs (Table 2). Overall, differences between platforms were smaller than differences between disease-associated alleles and null expectations.

**Table 2:** Disease-associated alleles from different genotyping platforms

	Mean frequency (disease-associated alleles)	Proportion ancestral (disease associated alleles)
**Manufacturer**		
Affymetrix (n = 638)	0.387	0.414
Illumina (n = 852)	0.395	0.444
Perlegen (n = 90)	0.410	0.434
Multiple platforms used (n = 430)	0.402	0.419
**SNPs genotyped in study**		
< 500,000 (n = 597)	0.399	0.452
500,00 to 1,000,000 (n = 205)	0.390	0.478
> 1,000,000 (n = 322)	0.390	0.388

Because many studies used multiple genotyping platforms, the number of disease-associated alleles by platform sums to greater than 1143. In 19 cases, the number of genotyped SNPs was unknown. Importantly, this table only describes the characteristics of disease-associated alleles.

### Estimated ages of SNPs

Genome-wide association studies were enriched for young SNPs compared to randomly selected HapMap loci (Figure 4B). Mean ages of SNPs were estimated to be 2.78 N_e _generations for HapMap loci and 2.74 N_e _generations for GWAS loci. However, the spread around the mean was quite large for each locus type. The probability densities in Figure 4B reveal that SNPs arising in the last 1 N_e _generations were over-represented in the GWAS dataset. This occurred because disease-associated alleles had an excess of low frequency derived alleles (the same sorts of alleles that tend to occur in young SNPs). Recall that these calculations assumed that SNPs were neutral. Directional selection would reduce the expected ages of SNPs for both types of loci [39]. Similarly, ascertainment bias due to the small sample size of the SNP discovery panel [43] can affect both types of SNPs.

### Odds ratios

**Figure 5:**
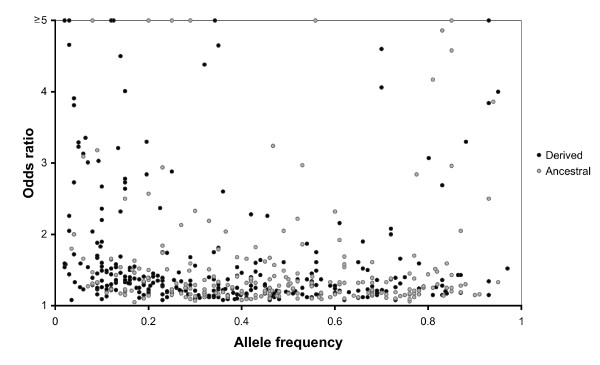
**Odds ratios for ancestral and derived alleles as a function of allele frequency**. Derived alleles are represented by black circles, and ancestral alleles are represented by grey circles. A total of 530 alleles have odds ratio data.

The findings of previous studies [1,13,16] were confirmed: most disease-associated alleles only increase disease risk by only a moderate amount (Figure 5). This is consistent with expectations from population genetics theory as alleles with high odds ratios are expected to have a higher fitness burden [44]. In addition, odds ratios of disease-associated alleles varied by frequency. The median odds ratio of ancestral alleles was 1.28 and the median odds ratio of derived alleles was 1.32. This indicates that the average effect of derived alleles was slightly larger than ancestral alleles (*p*-value < 0.05, Mann-Whitney U test). Low frequency alleles with high odds ratios tended to be derived alleles, but this was simply a byproduct of GWAS being enriched for derived alleles. Few intermediate frequency disease-associated alleles had high odds ratios. 26.8% of disease-associated SNPs with a minor allele frequency ≤ 0.2 had an odds ratio > 2, while only 8.0% of SNPs with a minor allele frequency > 0.2 had an odds ratio > 2 (*p*-value < 0.0001, Fisher's exact test). It is difficult to detect statistical associations between diseases and low frequency marker alleles, suggesting that the high odds ratios observed for SNPs with a minor allele frequency ≤ 0.2 were indicative of a "winner's curse." As only those alleles that are statistically significant were reported, published odds ratios may be overestimated [45].

## Discussion

Disease-associated alleles were more likely to be low frequency derived alleles than neutral and CEU HapMap expectations. Patterns were similar for alleles associated with different phenotypic classes. These findings were independent of publication date and genotyping platform. SNPs implicated in genome-wide association studies were enriched for young SNPs compared to randomly selected HapMap loci. One caveat is that that the majority of published studies to date have used european populations, and it is unclear whether these patterns will apply to other populations.

### Statistical power, sample sizes, and allele frequencies

Because statistical power is minimal at extreme allele frequencies, it is not surprising that most disease-associated alleles have minor allele frequencies greater than 0.1 (Figure 3). The relative inability of GWAS to explain the high heritabilities of many diseases [7] suggests that many genes responsible for common diseases might actually be at undetectably low frequencies. Simulations reveal that much of the fitness variance associated with genetic diseases can be due to very low frequency, large-effect alleles [46]. Theoretical work also indicates that rare causal alleles can create associations that are credited to common marker alleles (a phenomenon that has been called "synthetic association") [47]. Alternatively, common genetic diseases may be due to multiple allele of small effect, synergistic epistasis, and/or genotype-by-environment interactions [6]. To avoid only detecting associations with intermediate frequency alleles, larger sample sizes are needed. Increasing the number of genotyped SNPs also results in a greater likelihood of detecting an association [48], but all SNPs are not equally informative. In addition, the results of this study suggest that future GWAS may benefit from the inclusion of many young SNPs with low frequency derived alleles.

### Genetic background and linkage phase of causal and marker alleles

Genetic background and linkage phase may explain why disease-associated alleles were enriched for derived alleles. Consider the following thought experiment: Two alleles already segregate at a marker locus when a causal mutation occurs at a nearby locus. If the causal mutation occurs in an ancestral genetic background, only a small proportion of disease-associated marker alleles will be in phase with the causal mutation. This is because ancestral alleles tend to have higher frequencies than derived alleles [26]. As a result, the (*P*(*B*|*A*) - *P*(*B*|*a*))^2 ^term in Equation A1 (see Appendix) tends to be smaller when causal alleles are in phase with an ancestral allele at the marker locus. All other things being equal, linkage disequilibrium (*r*^2^) and statistical power are greater when causal mutations occur in a derived genetic background.

Recombination breaks down linkage disequilibrium between causal and marker alleles over time, reducing the likelihood of statistical associations. This is consistent with the finding that SNPs showing detectable associations with genetic disease are younger than randomly selected HapMap SNPs. Genome-wide association studies are also less likely to be successful if mutations occur multiple times at a causal locus. This is because the causal alleles can be found in multiple genetic backgrounds, reducing statistical associations between causal alleles and marker alleles. Population heterogeneity can also be an issue, as causal mutations can occur in different genetic backgrounds in different populations [49,50].

### Natural selection against disease alleles

Natural selection against deleterious alleles may also cause disease-associated alleles to differ from the rest of the genome. Population genetic theory indicates that marker alleles linked to low fitness causal alleles are expected to be uncommon [30,51]. This is in agreement with the finding that alleles associated with genetic disease tend to be found at lower frequencies than randomly selected HapMap alleles. Natural selection may also be able to explain differences in SNP age between disease-associated alleles and HapMap alleles.

The efficacy of selection varies for different genetic diseases. Because fitness refers to the expected contribution to the next generation's gene pool, diseases with late onset are likely to be found at higher frequencies. In addition, allele frequency distributions are shaped by the evolutionary history of a disease. Selection pressures that change over time can allow disease alleles to segregate at intermediate frequencies [52]. The genetic architecture of a disease also affects the strength of selection: Tajima's D and D_n_/D_s _ratios reveal that the signature of selection is stronger for Mendelian diseases than complex genetic diseases [53]. This may explain why different phenotypic classes have slightly different profiles (Table 1). However, alleles associated with morphological traits (as opposed to disease) differ from null expectations. Additional factors than natural selection may be required to explain why GWAS alleles differ from the rest of the genome.

### Can population size changes explain the observed patterns?

Disease-associated alleles are enriched for derived low frequency alleles, a pattern that can occur when populations increase in size [54,55]. However, population expansions affect the frequency distributions of all alleles, not just disease-associated alleles. Because HapMap data do not show an excess of derived low frequency alleles relative to neutral expectations (Figure 2), this indicates that population size changes alone cannot fully explain the characteristic patterns of disease-associated alleles.

## Conclusion

At these initial stages, alleles found via genome-wide association studies tend to be low hanging fruit. However, there is strong evidence that disease-associated alleles differ from the rest of the genome. Costs of microarray-based genotyping platforms are decreasing, and as the number of SNPs analyzed per individual increases, so too does the chance of detecting direct associations between disease and causal SNPs (rather than merely linked marker SNPs). In addition, direct sequencing is becoming increasingly affordable and it allows previously unknown SNPs to be identified. Because of this, direct sequencing of large genomic regions adjacent to disease-associated marker alleles is advisable. Direct sequencing of GWAS-informed regions can also be combined with familial inheritance patterns to improve genetic linkage analyses. This brings up an intriguing question: are causal alleles for a particular trait more likely to be ancestral or derived? Also, how can GWAS be planned to maximize the likelihood of detecting candidate genes associated with a particular disease? Combining the perspectives of genetic epidemiology and evolutionary genetics allows these questions to be answered.

## Competing interests

The author declares that they have no competing interests.

## Authors' contributions

JL conceived the study, participated in the analysis of the data, and wrote the manuscript.

## Appendix

Statistical power in GWAS is a function of odds ratios and the amount of linkage disequilibrium between causal alleles and marker alleles. The relevant measure of linkage disequilibrium in this case is *r*^2 ^[56]. Consider a causal locus with two segregating alleles (*A *and *a*), and a linked marker locus with two segregating alleles (*B *and *b*). *r*^2 ^is defined as:

(A1) $$r^{2}={\left(P(B|A)-P(B|a)\right)}^{2}\frac{y(1-y)}{x(1-x)},$$

where *P*(*B*|*A*) and *P*(*B*|*a*) are the probabilities that a haplotype has the marker allele *B *given a linked causal allele of *A *or *a*, *y *is the frequency of the disease allele at the causal locus, and *x *is the frequency of the disease-associated allele at the marker locus.

## Pre-publication history

The pre-publication history for this paper can be accessed here:

http://www.biomedcentral.com/1755-8794/3/57/prepub

## Supplementary Material

Additional file 1 **GWAS data**. This file is a Microsoft Excel spreadsheet that contains allele frequencies, ancestral vs. derived state, and phenotypic class for each disease-associated allele analyzed in this study. Click here for file
